# Diffusion Tensor Imaging Findings in Post-Concussion Syndrome Patients after Mild Traumatic Brain Injury: A Systematic Review

**DOI:** 10.3389/fneur.2016.00156

**Published:** 2016-09-19

**Authors:** Edrea Khong, Nicole Odenwald, Eyesha Hashim, Michael D. Cusimano

**Affiliations:** ^1^Department of Surgery, Division of Neurosurgery, Injury Prevention Research Office, Saint Michael’s Hospital, Toronto, ON, Canada

**Keywords:** diffusion tensor imaging, mild traumatic brain injury, post-concussion syndrome, biomarker, systematic review

## Abstract

**Objectives:**

To review the evidence for the use of diffusion tensor imaging (DTI) parameters in the human brain as a diagnostic tool for and predictor of post-concussion syndrome (PCS) after a mild traumatic brain injury (mTBI).

**Design:**

Systematic review.

**Data sources:**

All relevant studies in AMED, Embase, MEDLINE, Ovid, PubMed, Scopus, and Web of Science through 20 May, 2016.

**Study selection:**

Studies that analyze traditional DTI measures [fractional anisotropy (FA), mean diffusivity (MD), radial diffusivity (RD), and axial diffusivity (AD)] and the severity of PCS symptoms or the development of PCS in humans after an mTBI.

**Data extraction:**

Population studied, patient source, mTBI diagnosis method, PCS diagnosis method, DTI values measured, significant findings, and correlation between DTI findings and PCS.

**Data synthesis:**

Ten studies investigated correlations between DTI values and PCS symptom severity or between DTI values and the development of PCS in mTBI patients. Decreased FA and increased MD and RD were associated with the development and severity of PCS. AD was not found to change significantly. Brain regions found to have significant changes in DTI parameters varied from study to study, although the corpus callosum was most frequently cited as having abnormal DTI parameters in PCS patients.

**Conclusion:**

DTI abnormalities correlate with PCS incidence and symptom severity, as well as indicate an increased risk of developing PCS after mTBI. Abnormal DTI findings should prompt investigation of the syndrome to ensure optimal symptom management at the earliest stages. Currently, there is no consensus in the literature about the use of one DTI parameter in a specific region of the brain as a biomarker for PCS because no definite trends for DTI parameters in PCS subjects have been identified. Further research is required to establish a standard biomarker for PCS.

## Introduction

Traumatic brain injury (TBI) is an important global health issue, with the incidence of TBI reported to hospitals in developed countries being approximately 200 per 100,000 people annually (1). Globally, approximately 10 million TBIs are serious enough to result in death or hospitalization each year (2). TBI is more common in adolescents and young adults; a Canadian study found that 20% of students in grades 7–12 had sustained a TBI (3). Classification of TBI as a mild traumatic brain injury (mTBI) is primarily based on an initial Glasgow Coma Scale (GCS) score of 13–15 (4). Other mTBI classification criteria consider the duration of loss of consciousness (LOC) and duration of posttraumatic amnesia (PTA), if present (5). mTBIs are the most frequent TBIs, accounting for 70–90% of all brain injuries treated at hospitals. However, because a majority of mTBI cases are not reported to hospitals, the true incidence of mTBI is estimated to be above 600 per 100,000 people per year (6).

Post-concussion syndrome (PCS), also referred to as post-concussional disorder (PCD), refers to a set of somatic, affective, and cognitive symptoms that manifests days after the initial head injury. Although these symptoms usually resolve within 3 months, they can persist for longer (7). Patients whose symptoms persist for less than 3 months are referred to as having experienced post-concussion symptoms, while those with symptoms persisting for longer than 3 months are diagnosed with PCS (8, 9). PCS often has a significant impact on quality-of-life, but currently there are no validated treatments for PCS beyond patient monitoring and symptom management.

The first step toward developing an effective treatment is to understand the pathophysiology and anatomical basis of the development of PCS and establish dependable biomarkers of the syndrome. Unfortunately, the current definition of PCS is vague because as a *syndrome*, it is only a set of signs and symptoms. Diagnosing PCS depends solely on clinical criteria, the judgment of the physician or healthcare professional, the patient’s self-reporting of symptoms, and the diagnostic assessment selected. For this reason, PCS diagnosis is unreliable and poorly defined.

The Rivermead Post-Concussion Symptoms Questionnaire (RPCSQ) (10) is often used to quantify PCS symptoms. However, it has been shown that these criteria do not meet modern psychometric standards, and it was suggested that the usual practice of summating the RPCSQ into a single score is unreliable (11). Other assessments used in the diagnosis of PCS include the World Health Organization (WHO) International Classification of Diseases (ICD) guidelines (12), the British Columbia Postconcussion Symptom Inventory (BC-PSI) (13), and the Neurobehavioral Symptom Inventory (NSI) (14). Unfortunately, PCS symptoms are not specific to TBI patients (7, 15–18), further complicating PCS diagnosis *via* these assessments. mTBI patients have been found to report a greater number and increased severity of PCS symptoms when compared with moderate or severe TBI patients (19, 20). Identifying a biomarker specific to TBI patients with PCS would greatly improve diagnosis and treatment.

Axonal damage can cause impaired network function (21, 22) and may explain the symptoms experienced by patients after a TBI (23). Diffusion tensor imaging (DTI) is a non-invasive, *in vivo* imaging technique that measures the quantity and direction of water molecule diffusion (24). It is well-documented and validated for use in mapping microstructural changes, such as axonal damage, in the brain (24–28). The most commonly measured DTI parameter in brain research is fractional anisotropy (FA) (28), a measure of the directionality of diffusion (25). Other common DTI parameters include mean diffusivity (MD), a scalar measure of the total diffusion within a voxel (25), radial diffusivity (RD), a scalar measure of the diffusion in two directions perpendicular to the length of an axon, and axial diffusivity (AD), a scalar measure of the diffusion along the length of an axon (29). Through these measurements, DTI can detect microstructural changes in the brain’s white matter tracts; abnormalities in these measurements indicate axonal damage, which may correlate with PCS symptoms (28, 30).

Diffusion tensor imaging has been used in the PCS population to study axonal damage that may be the underlying cause of the syndrome. A specific DTI biomarker for PCS would help identify and characterize these patients, providing the basis for treatments that target the anatomical deficits that cause the syndrome. A number of studies have looked at the classic DTI parameters in the PCS population (31–40). These studies offer information that can be helpful for clinicians and patients managing PCS, but often differ in the DTI parameters analyzed and in the brain regions found to have abnormal DTI values. A review to summate the current literature will help to design future studies to address gaps in the field. This paper reviews the use of DTI parameters as biomarkers for diagnosing and predicting PCS after mTBI. By summarizing the current literature on the use of DTI parameters in patients with PCS after an mTBI, this paper aims to assist future researchers, clinicians, and patients in determining the role of DTI as a diagnostic tool for and predictor of PCS.

## Methods

### Literature Search

A comprehensive literature search was conducted on AMED, Embase, MEDLINE, Ovid, PubMed, Scopus, and Web of Science for all relevant articles reporting on the use of DTI in subjects who developed PCS post-mTBI, through 20 May, 2016. The databases were searched with the following search phrase using the Boolean logic operators “OR” and “AND”: “(DTI OR diffusion tensor imaging OR diffusion tractography) AND (mTBI OR mild traumatic brain injury OR concussion) AND (postconcussive syndrome OR post-concussive syndrome OR post concussive syndrome OR postconcussion syndrome OR post-concussion syndrome OR post concussion syndrome) AND human.” To ensure maximal article capture, these search terms also encompassed the following Medical Subject Headings (MeSH) terms: “diffusion tensor imaging,” “brain injuries,” and “post-concussion syndrome.” Manual searching of relevant journals and reference lists of studies found in the above search provided additional articles.

**Figure 1:**
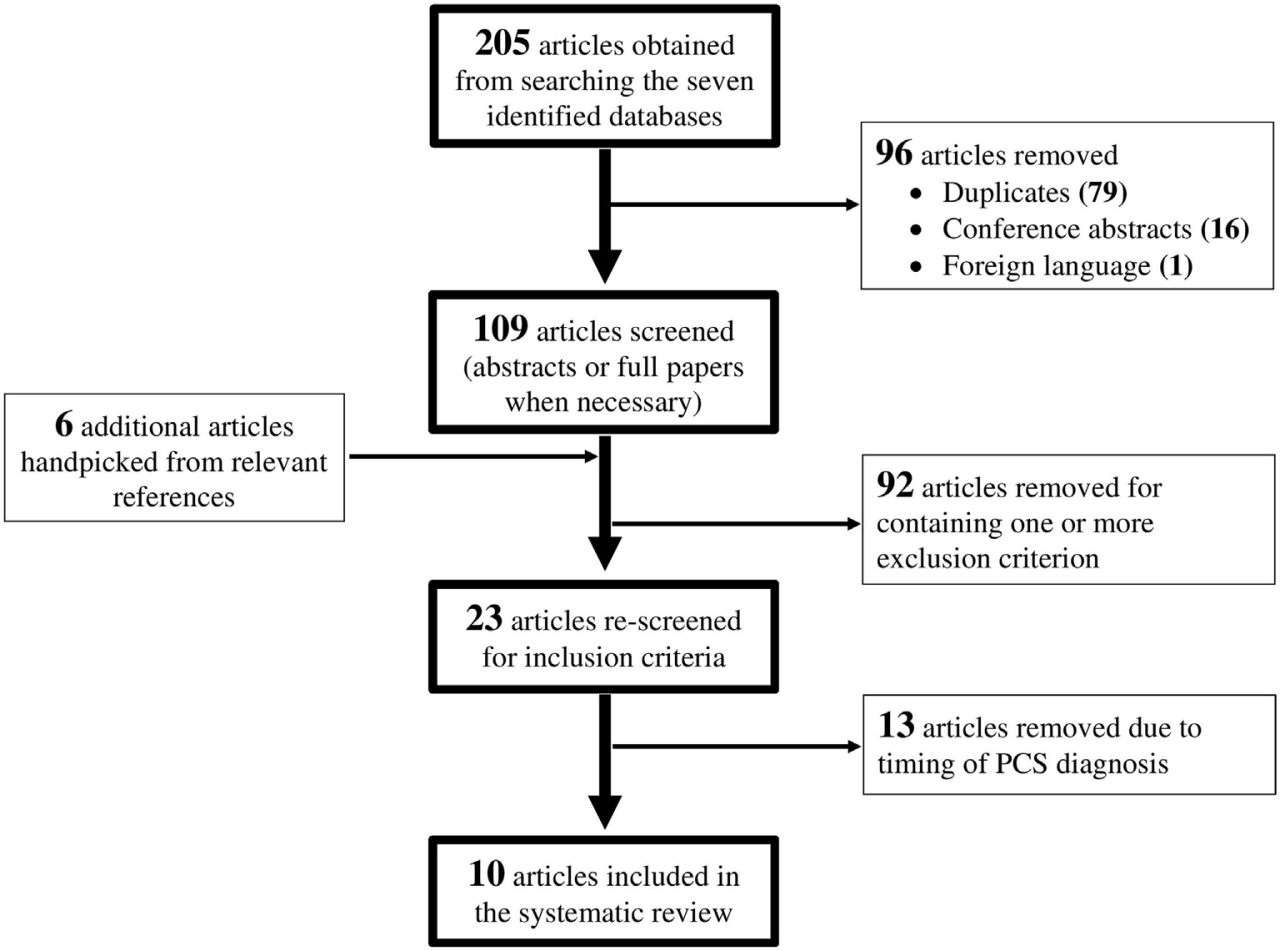
**Flow chart depicting the paper selection process**.

The search terms identified above yielded 205 studies. For this review, the PCS population was defined as patients who experience persistent symptoms for 3 months or longer post-injury. Inclusion criteria were studies published in English; use of human participants; and studies that analyzed changes in measured DTI parameters in patients with PCS following an mTBI. Exclusion criteria were studies that did not report method of diagnosing PCS; studies that did not report method of diagnosing mTBI; studies that were not original research; and studies with fewer than six participants. Two readers independently screened all 205 studies, removing 79 duplicates, 16 conference abstracts, and 1 foreign language paper. Six additional studies were identified from relevant reference lists and journals. Of the 115 studies remaining, 92 were excluded for containing one or more exclusion criterion. A further 13 studies were removed after rescreening because they included patients who were assessed for PCS symptoms within 3 months of injury. This resulted in 10 studies that were included in this review. The flow diagram for the paper selection process is presented in Figure 1.

### Data Extraction and Analysis

Each study was assessed by two reviewers for quality using STROBE guidelines (41). A point was awarded for each required criterion that was met. The two reviewers performed individual assessments, and scores were compared, with discrepancies resolved by discussion. The two reviewers also performed data extraction for the population studied, patient source, number of PCS patients, patient demographics (age and gender), mechanism of injury, mTBI diagnosis method, PCS diagnosis method, time interval between injury and PCS diagnosis, time interval between injury and imaging, control group characteristics (screening process, number, and matching process), DTI values measured, DTI analysis method [voxel-wise or region of interest (ROI)], significant findings, conclusions (correlations between DTI and PCS), and study limitations for all nine studies. DTI studies most often do not quantify changes in DTI parameters because there are no established values for healthy patients, so only qualitative changes (increases and decreases) in these parameters were extracted for this review. Results of data extraction were compared between the reviewers, with discrepancies resolved with help from a third reader.

## Results

A total of 205 studies were screened for eligibility, with 10 studies, published within the last 6 years, qualifying for review based on the inclusion and exclusion criteria. All studies scored between 17 and 20 points out of a possible 22 in STROBE quality assessment. The 10 studies included PCS patients from 8 to 65 years of age, with the mean age across all studies being 29.58 years. One study analyzed a pediatric population (31), one study analyzed a mixed pediatric and adult population (35), and the rest analyzed adult populations. Also, 235 PCS patients were included in the 10 studies reviewed, with 141 male and 44 female PCS patients tested in the 8 studies that reported sex breakdown. All studies included control groups. One study (36) used a combination of patients with orthopedic injuries and healthy patients as controls, two studies (34, 39) used uninjured military members as controls, and the remaining seven studies used healthy controls. Nine studies (31–36, 38–40) performed a group-wise comparison between controls and PCS subjects, while the last study (37) directly matched PCS patients to controls. Matching was achieved on the bases of age, gender, and years of education.

For the DTI analysis, four studies (33, 38–40) performed a whole brain voxel-wise analysis, four studies (31, 32, 34–36) used an ROI analysis, and two studies (37) used a voxel-wise approach to identify ROIs for subsequent ROI analysis. In the 10 studies reviewed, the time interval between injury and imaging ranged from 7 days to 259 months, with a median imaging time of 20.5 months post-injury. The time interval between injury and PCS assessment ranged from 3 to 259 months, with a median assessment time of 23.2 months post-injury.

Seven of the ten studies analyzed DTI parameters in imaging conducted after PCS was diagnosed, thereby assessing the value of DTI as a biomarker of PCS (Table 1). The remaining three studies analyzed imaging conducted at the subacute or pre-PCS stage of injury in patients who were later diagnosed with PCS (Table 2). The importance of these studies is in the prediction of PCS development in mTBI patients.

**Table 1:** **Studies looking at DTI parameters as a biomarker for PCS**.

Article	Population studied	Diagnosis method (mTBI; PCS)	DTI analysis approach	DTI values measured
Bartnik-Olson et al. (31)	Pediatric patients, sustained a sports-related mTBI in an organized athletic event	International Conference on Concussion in Sport; self-reported symptoms	ROI analysis	FA, MD, RD, AD
Bouix et al. (32)	Patients with persistent PCS, sustained an mTBI in an MVC, blast exposure, sports-related event, or assault	Emergency department triage; headaches, emotional dysregulation, cognitive, or memory impairments	ROI analysis	FA, MD, RD, AD
Dean et al. (33)	Patients who sustained an mTBI but did not report to the hospital, no reported mechanism of injury	WHO (ICD-10); RPCSQ	Voxel-wise analysis	FA
Delano-Wood et al. (34)	Military veterans with a closed head TBI from blast exposure or blunt force trauma	US DoD and the Department of Veterans Affairs TBI Task Force; NSI	ROI analysis	FA
Levin et al. (36)	Post-deployment veterans and service members, sustained mTBI in a blast exposure	Physician diagnosis; NSI	ROI analysis	FA, MD
Maruta et al. (37)	Patients with a single, isolated concussive injury to the head, no reported mechanism of injury	Physician diagnosis; self-reported symptoms	Voxel-wise and ROI analysis	FA, MD, RD, AD
Miller et al. (39)	Military veterans, sustained an mTBI in a blast exposure	American Congress of Rehabilitation Medicine; RPCSQ	Voxel-wise analysis	FA

**Table 2:** **Studies looking at DTI parameters in prospective PCS patients**.

Article	Population studied	Diagnosis method (mTBI; PCS)	DTI analysis approach	DTI values measured
D’Souza et al. (35)	Patients from a neurosurgery clinic, no reported mechanism of injury	American Congress of Rehabilitation Medicine; RPCSQ	ROI analysis	FA, MD
Messé et al. (38)	Patients presenting to the emergency department, sustained an mTBI in an MVC, pedestrian injury, or aggression incident	American Congress of Rehabilitation Medicine; self-reported symptoms	Voxel-wise analysis	MD
Polak et al. (40)	PCS patients referred from a concussion clinic, sustained an mTBI in a sports-related event, fall, or when struck by an object	Physician diagnosis *via* the Buffalo Concussion Treadmill Test; WHO	Voxel-wise analysis	FA, MD, RD, AD

A decrease in FA and increase in MD and RD were commonly observed in PCS patients post-mTBI. The most common finding across all studies was that FA decreased in patients with PCS following mTBI compared to controls, although three studies included in the review found no significant changes in FA (Table 3). An increase in MD was also found, although three of the six studies that analyzed MD did not find a significant change (Table 4). Messé et al. (38) compared mTBI patients with PCS to mTBI patients not experiencing PCS and found higher MD values in the PCS-present group. An increase in RD was also found in three of five studies that analyzed RD (Table 5). No changes in AD were observed in the five studies that analyzed this parameter. These changes in DTI parameters also had a positive correlation with PCS symptom severity. The corpus callosum was most frequently reported as being affected in PCS, with reduced FA and increased MD and RD (Table 6).

**Table 3:** **Studies that analyzed fractional anisotropy**.

Article	Affected region	Change
**Studies that found changes in FA**		
Bouix et al. (32)	Whole brain	↓
Dean et al. (33)	Right anterior corona radiata, internal capsule (anterior limb), corpus callosum (splenium), fornix, frontal medial superior gyrus	↓
Delano-Wood et al. (34)	Pontine tegmentum	↓
D’Souza et al. (35)	Corpus callosum, left uncinate fasciculus, bilateral superior thalamic radiations	↓
Levin et al. (36)	Corpus callosum	↓
Miller et al. (39)	Greater number of white matter clusters	↓
Polak et al. (40)	Corpus callosum (genu)	↓
**Studies that found no changes in FA**		
Bartnik-Olson et al. (31)		
Maruta et al. (37)		
Messé et al. (38)		

**Table 4:** **Studies that analyzed mean diffusivity**.

Article	Brain region	Change
**Studies that found changes in MD**		
Bartnik-Olson et al. (31)	Corpus callosum (genu)	↑
D’Souza et al. (35)	Left uncinate fasciculus	↑
Messé et al. (38)	Corpus callosum (forceps major and minor), inferior fronto-occipital fasciculus, inferior longitudinal fasciculus, superior longitudinal fasciculus, corticospinal tract, left anterior thalamic radiation	↑
Polak et al. (40)	Corpus callosum (genu)	↑
**Studies that found no changes in MD**		
Bouix et al. (32)		
Levin et al. (36)		
Maruta et al. (37)		

**Table 5:** **Studies that analyzed radial diffusivity**.

Article	Brain region	Change
**Studies that found changes in RD**		
Bartnik-Olson et al. (31)	Internal capsule (right anterior limb)	↑
Bouix et al. (32)	Whole brain	↑
Polak et al. (40)	Corpus callosum (genu)	↑
**Studies that found no changes in RD**		
Maruta et al. (37)		
Messé et al. (38)		

**Table 6:** **Studies that found changes in the corpus callosum**.

Specific region	DTI value	Change	Article
Genu	MD	↑	Bartnik-Olson et al. (31)
			Polak et al. (40)
	RD	↑	Polak et al. (40)
Splenium	FA	↓	Dean et al. (33)
Whole corpus callosum	FA	↓	Levin et al. (36)
			D’Souza et al. (35)
Forceps major	MD	↑	Messé et al. (38)
Forceps minor	MD	↑	Messé et al. (38)

## Discussion

### Findings in DTI Parameters

Decreased FA and increased MD and RD were reported in PCS patients compared to healthy controls or PCS-absent trauma patients. Abnormal DTI values were also reported to be correlated with an increase in number and severity of PCS symptoms, suggesting that greater axonal damage causes more severe PCS symptoms. Decreased FA is a well-documented finding in brain injury (42–46), as is increased MD (42, 44, 45) and RD (43). In addition, regions within the corpus callosum were most often found to be affected in PCS patients. Two additional articles reporting on case studies also found decreased FA, specifically in PCS patients (47, 48). The corpus callosum is involved in inter-hemispheric integration of motor, sensory, and cognitive information, and damage to this area might lead to extensive behavioral, emotional, and cognitive impairments, as observed in the PCS groups. Although the effects of damage to the corpus callosum are not fully understood, it is reasonable to expect that these effects would include the symptoms of PCS patients.

Studies included in this review assessed different patient populations, including pediatric, adult, and military. Although age- and education-related differences in structural and functional neuroanatomy have been documented (49, 50), all of the studies had a case–control design. Patients were matched to controls by age, gender, and education level, therefore ensuring that changes detected in DTI parameters were not a result of comparisons made between inherently distinct populations. However, consistent findings in FA, MD, and RD across the studies suggest that the microstructural white matter damage detected is consistent in all patient groups. This possibility increases the value of DTI as a universal biomarker of PCS. In addition, 8 of the 10 studies (31–35, 38–40) excluded patients with abnormal CT or routine clinical MRI findings. This exclusion criterion indicates that there is damage identified by DTI, which is not detected *via* more commonly used modalities, further emphasizing the utility and importance of DTI in the clinical setting. It is often acknowledged that the more widely available structural neuroimaging modalities have limited value in the mTBI and PCS populations (8, 51, 52).

Post-concussion syndrome has been found to be more prevalent in the mTBI population than the moderate or severe TBI populations (19, 20). However, it has been noted that axonal injury in these more severely injured groups usually presents with focal lesions and so is detectable *via* clinical MRI (53). Therefore, not all axonal injury is indicative of PCS. This evidence suggests that generalized axonal injury does not correlate well with PCS, but diffuse axonal injury (DAI), which presents as widespread axonal injury limited to the microstructure scale, may be more specific to PCS. This is also supported by a recent study using magnetic resonance spectroscopy (MRS), which found significant correlations between DAI and PCS (54). However, PCS may not only be a result of neurological damage but may also develop due to psychological distress (55–57). For this reason, a patient who has not sustained an mTBI may still experience PCS because it is not unique to the mTBI or TBI population. Although patients with DAI may develop PCS, not all PCS patients have DAI.

### ROI versus Voxel-Wise Analysis

Two studies (37, 39) that applied a voxel-wise analysis of the whole brain reported no significant findings in the corpus callosum. Maruta et al. (37) reported no significant findings in any brain region but was the only study to have no significant findings in all four DTI parameters in a whole brain analysis. Miller et al. (39) did find a decreased FA in several white matter clusters in the whole brain but did not identify specific brain regions where the clusters were located. Voxel-wise analysis requires intersubject registration of subjects’ brains and normalization to standard atlases. Both of these processes involve smoothing and hence may result in masking small differences between subjects or groups. All studies that used ROI analysis found significant changes in DTI parameters, suggesting that ROI analysis might be more proficient at identifying changes in specific brain regions. However, ROI analysis has some limitations: it requires a brain structure to be predefined for analysis, and manual selection of the region to draw an ROI may lead to intersubject differences in ROI location. Despite these limitations, the success of ROI analysis in the studies reviewed is encouraging; once a particular region has been identified as most commonly damaged in PCS, diagnosis *via* DTI will be faster than if a voxel-wise approach was necessary.

### Data Synthesis

Although DTI can detect axonal damage and possibly predict PCS onset or be used to diagnose PCS, there is insufficient evidence supporting the observed results to validate any parameter in a specific brain region as a biomarker for PCS. The trends in the parameters are a decrease in FA and an increase in MD and RD, but there is no uniformity in the brain areas investigated for these changes. These findings suggest that there may be more than one DTI biomarker for PCS and that axonal damage does contribute to PCS symptoms. The subjective nature of PCS and the possibility of a large number of brain regions being involved in PCS may have led to the indefinite results. Each patient has a unique illness experience due to their baseline for many of the symptoms, such as fatigue, feelings of depression, feelings of frustration, forgetfulness, poor concentration, and restlessness. Patients have varying pain tolerances and emotional fortitudes that could either magnify or diminish the severity of PCS symptoms being reported. In addition, a researcher may focus on one or more brain regions or on the entire brain, depending on his or her research interests and image processing preferences.

### Other Potential PCS Biomarkers

Other imaging modalities have been used to study PCS. In addition to the poor value of CT and MRI in the PCS population, studies have shown conflicting results in positron emission tomography (PET) in the PCS population, with some finding correlations between PET results and PCS (58–60), while others do not (51). A more promising imaging modality may be MRS, which has been used to detect DAI that is significantly correlated with PCS (54), although there is unsubstantial evidence that it is a reliable PCS biomarker. Evoked potential (EP) studies have concluded that significant results in the EP data correlate with PCS (61, 62), but there is not enough concrete evidence to support these measurements as a PCS biomarker. Biochemical markers of PCS may also be viable. A review paper (63) identified S100 proteins, neuron-specific enolase (NSE), and cleaved Tau protein (CTP) as potential serum biochemical markers for predicting PCS after an mTBI. The review concluded that none of the three compounds are well-validated for use, although S100 was most widely studied in the mTBI population and appears to be the most promising. It is possible that a combination of DTI, clinical factors, and biochemical markers may be needed to accurately and objectively diagnose PCS or predict its development after an mTBI.

### Future Research Directions

Further research into this topic is necessary. All of the studies included in this review are cross-sectional. Future studies should consider a longitudinal cohort study design to track changes in DTI parameters during PCS progression and resolution, which would provide more concrete evidence of a specific biomarker for PCS. Recruiting a larger PCS patient population is required to reduce sample size biases. Research on DTI parameters in the brain is also required to establish “normal” values for FA, MD, RD, and AD, so that significant differences are based on a universal standard as opposed to being derived from each study’s controls. This will ensure that significant results are not misrepresented. Future case–control studies should try to use an orthopedic or other non-head trauma control group to help remove error in PCS reporting due to factors other than the sustained mTBI. Case–control studies may also consider comparing PCS-present mTBI patients to PCS-absent mTBI patients to eliminate most external confounding factors. These studies would be more likely to observe changes in DTI parameters specifically due to PCS. The large range of time intervals between injury and imaging reported in the ten reviewed articles is also a concern. In addition, 13 articles were excluded from this review because researchers conducted PCS assessment within 3 months of injury. It is suggested that future studies include patients whose PCS symptoms persist for 3 months or longer, as the literature supports PCS diagnosis when symptoms persist for this length of time. Finally, a standard PCS assessment should be administered to ensure consistency across all future studies. Consulting the Common Data Elements identified by the National Institutes of Health would help establish assessment standards.

## Conclusion

To our knowledge, this is the first systematic review that examines the use of DTI parameters in the human brain as a diagnostic tool for patients with PCS and a predictor of PCS in mTBI patients. DTI abnormalities indicate axonal damage, which leads to an increased risk of developing PCS after an mTBI. However, no DTI biomarker for PCS is identified due to the small body of research conducted on the topic and the heterogeneity of results reported. Further research is required to establish a standard DTI biomarker for PCS diagnosis and prediction.

## Author Contributions

EK and NO – study concept, study design, literature search, quality assessment, data extraction and analysis, manuscript draft, manuscript critical revision, and final approval. EH and MC – study concept, study design, manuscript critical revision, and final approval. All authors agree to be accountable for all aspects of the work.

## Conflict of Interest Statement

The authors declare that the research was conducted in the absence of any commercial or financial relationships that could be construed as a potential conflict of interest.
